# Effect of multimodal therapy in the treatment of adenocarcinoma of the esophagus and gastroesophageal junction : a population-based analysis from the German Clinical Cancer Registry Group

**DOI:** 10.1007/s00423-026-04066-7

**Published:** 2026-05-29

**Authors:** Thaer S. A. Abdalla, Markus Kist, Ruslan Arify, Monika Klinkhammer-Schalke, Sylke Ruth Zeissig, Kees Kleihues-van Tol, Stefan Benz, Micheal Schweigert, Stanislav Litkevych, Ahmed S. A. Abdalla, Markus Zimmermann, Stephan Kersting, Justus Baecker, Ulrich Friedrich Wellner, Tobias Keck, Michael Thomaschewski, Richard Hummel

**Affiliations:** 1 Department of Surgery, Univesity of Luebeck, Ratzeburger Allee 160, Lübeck, 23562 Germany; 2 German Cancer Registry Group of the Society of German Tumor Centers—Network for Care, Quality and Research in Oncology (ADT), Berlin, 14057 Germany; 3 Institute of Clinical Epidemiology and Biometry (ICE-B), University of Würzburg, Würzburg, 97080 Germany; 4 Bavarian Cancer Registry, Bavarian Food and Health Safety Agency, Würzburg, Germany; 5 Department of Surgery, University Medical Center Greiswald, Ferdinand-Sauerbach-Strasse, Greifswald, 17475 Germany; 6 Department of General-, Visceral-, Thoracic- and Pediatric Surgery, Southwest Clinic Network, Calwer Strasse 68, Böblingen, 71034 Germany

**Keywords:** Multimodal therapy, Esophagus adenocarcinoma, Gastroesophageal junction tumor, Perioperative chemotherapy, Radiochemotherapy.

## Abstract

**Purpose:**

Multimodal treatment regimes combining surgery with perioperative chemotherapy and/or preoperative radiochemotherapy have become common treatment-modalities in resectable esophageal adenocarcinoma with controversial effects on oncological outcome.

**Methods:**

This population-based analysis was derived from 24 German cancer registries from 2000 to 2018. The overall survival was compared over three time periods, 2000-2006: pre-neoadjuvant era (PRE-NEO); 2007-2012: implementation of neoadjuvant treatment (NEO); 2013-2018: neoadjuvant/multimodal treatment strategies (POST-NEO).

**Results:**

For patients with adenocarcinoma of the esophagus (EAC) and gastroesophageal junction (GEJ), cTNM staging was documented in 9738 patients: Stage I (n=1405 (14%)), Stage II (n=1360 (14%)); Stage III (n=2680 (28%)); Stage IV (n=4293 (44%). The overall survival improved steadily over time for GEJ and EAC. Patients with Stage IV disease decreased over time in both tumor locations (GEJ: 71% PRENEO; 40% POST-NEO; EAC: 48% PRE-NEO to 34 %POST-NEO. Curative intended therapy increased over time (GEJ: 23% PRE-NEO; 50% POST-NEO; EAC: 29% PRENEO to 52% POST-NEO). In cTNM Stage II, perioperative chemotherapy and neoadjuvant radio chemotherapy followed by surgery were associated with longer overall survival in GEJ but not EAC compared to surgery alone. In Stage III, both multimodal treatment approaches were associated with longer overall survival in both EAC and GEJ patients when compared to surgery alone. Despite an obvious trend toward perioperative therapy, there was no statistical superiority between perioperative chemotherapy or neoadjuvant radiochemotherapy.

**Conclusion:**

Multimodal therapy was independently associated with improved overall survival, particularly in patients with cTNM Stage III disease and Stage II GEJ tumors. These findings support current guideline-based treatment strategies and underscore the relevance of multimodal care in appropriately selected patients.

**Supplementary Information:**

The online version contains supplementary material available at 10.1007/s00423-026-04066-7.

## Introduction

Over the last two decades, the management of esophageal adenocarcinoma (EAC) and gastroesophageal junction adenocarcinoma (GEJ) has undergone significant transformations, reflecting a dynamic and constantly evolving area in surgical oncology. EAC and GEJ adenocarcinoma represent malignant tumors arising from the glandular cells. The incidence of these aggressive tumors has shown a remarkable increase over the last twenty years, making them one of the most rapidly rising malignancies in Europe and North America [1, 2].

The advancements in diagnostic techniques, the better understanding of the disease biology, and the emergence of novel therapeutic modalities have contributed to substantial progress in the treatment of this devastating disease. According the CONCORD-3-Trial, an international study from 60 countries, the 5-year survival rate for EAC and GEJ adenocarcinoma between 2000 and 2014 ranges between 5 and 20% [3].

Till now, surgical resection remains the cornerstone of curative treatment. Regarding the surgical techniques, minimally invasive surgery has gained prominence over the past two decades. These advancements have not only improved postoperative outcomes but also expanded the pool of patients eligible for potentially curative surgical resection [4].

Furthermore, the incorporation of multimodal treatment approaches has become increasingly commonplace. Neoadjuvant chemoradiotherapy followed by esophagectomy has gained popularity in Western countries. However, perioperative chemotherapy also finds global support, leading to ongoing debates about the superiority of one approach over the other [5]. For instance, the CROSS protocol, involving neoadjuvant chemoradiotherapy followed by surgery, has emerged as a prominent treatment strategy for locally advanced disease in the Netherlands, while perioperative chemotherapy utilizing the FLOT protocol has gained popularity in Germany as the main neoadjuvant strategy for locally advanced EAC or GEJ [6]. The ESOPEC-trial is currently investigating the optimal treatment strategy for EAC and GEJ adenocarcinoma [5].

Given the differences in healthcare protocols and racial disparities among countries, oncological outcomes are significantly influenced by regional factors. Therefore, comprehensive information regarding local demographics, current treatment strategies, and survival outcomes are essential for adjusting guidelines to suit individual countries. Consequently, we conducted the present study to provide a representative overall picture of daily clinical practice in Germany. This study employed a population-based analysis utilizing data from 24 clinical cancer registries in Germany, provided by the Society of German Tumor Centers (ADT).

## Materials and methods

### Study design and database

Our study is derived from the German Cancer Registry Group of the Society of German Tumor Centers - (ADT). Data from 24 clinical registries from the years 2000 to 2018 was used according to the regulations of the German Cancer Registry Group. This study was approved by the local ethics committee of the University of Lübeck, Germany, #20–237. Of all patients with malignancies of the esophagus (codes C.15.0–9, ICD-O 3. edition (ICD-O-3)) or cardia (C.16.0, ICD-O 3. edition (ICD-O-3)), only patients with adenocarcinoma (ICD-O-3 code 8140-3) were included [6].

The following information was retrieved from the registry: sex (male vs. female), age at diagnosis (years), lymph node metastases (cN0, cN+), cT-stage (T1–T4), tumor location (upper third, middle third, lower third or cardia), the treatment intention (curative or palliative), general treatment modality (primary resection, multimodal therapy, definitive radiochemotherapy and palliative therapy), type of multimodal therapy (perioperative chemotherapy, neoadjuvant radiochemotherapy, primary surgery followed by adjuvant chemotherapy and chemotherapy alone followed by surgery), follow-up time (months after diagnosis), status at last follow-up (dead, alive).

cTNM staging was according to 8th edition AJCC/UICC staging for adenocarcinoma of the esophagus. UICC Stage I included cT1 N0 M0. UICC Stage II included cT1 N1 M0 and cT2 N0 M0, UICC Stage III cT2 cN1 M0 and cT3-4 N0-1 M0. UICC Stage IV included cT1-4 N2-3 M0, cT4b N0-2 M0 or any M1.

In order to analyse trends of treatment and their impact on outcome over time, we established based on the timepoint of clinical implementation of multimodal/neoadjuvant strategies around the years 2005/2006 three time periods for analysis: 2000–2006: pre-neoadjuvant era (PRE-NEO); 2007–2012: era of implementation of neoadjuvant treatment (NEO); 2013–2018: era of established neoadjuvant/multimodal treatment strategies (POST-NEO).

### Statistical methods

For statistical analysis, SPSS 29 for Windows (Armonk, NY, USA) was used. Descriptive statistics were used to describe patient baseline characteristics. To evaluate categorical variables, the Chi-Square test was applied. Survival curves for disease-free and overall survival of the patients were plotted using the Kaplan–Meier method and compared using the log-rank test. Results were presented as median survival in months.

The overall survival was computed as the period from the date of diagnosis to either the date of death or the last follow-up, whichever occurred first. For all statistical analyses, a p-value of *p* ≤ 0.050 was considered significant.

To assess the potential impact of missing staging data, a sensitivity analysis was performed comparing overall survival between patients with and without documented cTNM staging across the predefined time periods (PRE-NEO, NEO, POST-NEO) using Kaplan–Meier analysis and log-rank testing.

Additionally, multivariable Cox proportional hazards regression was performed adjusting for age group, sex, tumor location, clinical stage, treatment category, and treatment era. Hazard ratios (HR) with 95% confidence intervals (CI) were calculated.

## Results

### Patient characteristics and epidemiology

Clinical data on 24.729 patients with adenocarcinoma of the esophagus (EAC) and gastroesophageal junction (GEJ) were derived from 24 clinical cancer registries provided by the German Cancer Registry Group of the Society of German Tumor Centers - (ADT) diagnosed between 2000 and 2018. Overall the frequency of reported cases increased overtime for both tumor entities (Fig. S1). In this study population most of the patients were male (80%). This tendency was shown for both tumor entities (EAC male: 85%; GEJ male: 76%) (Fig. S2). Median age was 68 years ± IQR 17. For both tumor entities more patients were diagnosed > 65 years (EAC > 65 years: 58%; GEJ > 65%: 64%).

The cohort was divided into three time periods PRE-NEO (2000–2006), NEO (2007–2012) and POST-NEO (2013–2018), which included 7212, 8372 and 9145 patients, respectively. There was a similar distribution among the tumor entities over the time periods. cTNM was recorded in 9738 patients. Tumors were diagnosed as UICC stage I / II / III / IV disease in 14% / 14% / 28% / 44% of cases, with locally advanced and metastatic disease (stage III and IV) accounting for 72% of all cases. Tumors located at the GEJ presented with more advanced stages III/IV (78%) compared to EAC (64%), which showed a more even distribution among UICC stages at the time of diagnosis (Table 1).

**Table 1:** Patient characteristics

Variables	all *n* (% of total)	EAC *n* (% of total)	GEJ *n* (% of total)
Age (*n* = 24729)			
> 65 years	15,178 (61%)	5886 (58%)	9292 (64%)
< 65 years	9551 (39%)	4332 (42%)	5219 (36%)
Sex (*n* = 24729)			
Female	5058 (20%)	1567 (15%)	3491 (24%)
Male	19,671 (80%)	8654 (85%)	11,017 (76%)
Time Period (*n* = 24729)			
PRE-NEO (2000–2006)	7212 (29%)	2967 (29%)	4245 (29%)
NEO (2007–2012)	8372 (34%)	3519 (34%)	4853 (34%)
POST-NEO (2013–2018)	9145 (37%)	3731 (37%)	5414 (37%)
UICC Stage (*n* = 9738)			
Stage I	1405 (14%)	902 (21%)	503 (9%)
Stage II	1360 (14%)	638 (15%)	722 (13%)
Stage III	2680 (28%)	1220 (29%)	1460 (27%)
Stage IV	4293 (44%)	1503 (35%)	2790 (51%)
Treatment modalitiy (*n* = 6071)			
Primary Operation	1038 (17%)	344 (14%)	694 (19%)
Multimodal Therapy	1446 (23%)	608 (27%)	838 (22%)
Definitive Radiochemotherapy	279 (4%)	203 (9%)	76 (2%)
Palliative	3308 (56%)	1139 (50%)	2169 (57%)

Overall, the most frequent general treatment modality was palliative treatment (56%), followed by multimodal therapy (23%) and primary surgery (17%). Lastly, definitive radiochemotherapy represented the least frequent therapy modality (4%). There were no major differences found between the tumor entities for the treatment modalities (Table 1).

Furthermore, the overall survival for EAC and GEJ in stages I to IV is shown in Table 2. As expected, survival rates decreased in both tumor entities with increasing UICC stages. For example, 5-year survival decreased from 75% in UICC stage I down to 4% in UICC stage IV in EAC, and from 48% in UICC stage I down to 4% in UICC stage IV in GEJ. Results of the sensitivity analysis are presented in (Fig. S5).

**Table 2:** Overall survival for EAC and GEJ for 1,3 and 5 years according toUICC stages

Table a. Overall survival for EAC to cTNM			
	1-year OS in	3-yearsOS	5-years OS
Stage I	94%	83%	75%
Stage II	77%	44%	31%
Stage III	68%	31%	22%
Stage IV	39%	7%	4%
Table b. Overall survival for GEJ to cTNM			
	1-year OS	3-years OS	5-years OS
Stage I	80%	59%	48%
Stage II	74%	43%	30%
Stage III	70%	35%	25%
Stage IV	35%	7%	4%

UICC according to the TNM 8th Classification; p<0.05

### Temporal trends of overall survival

**Fig. 1:**
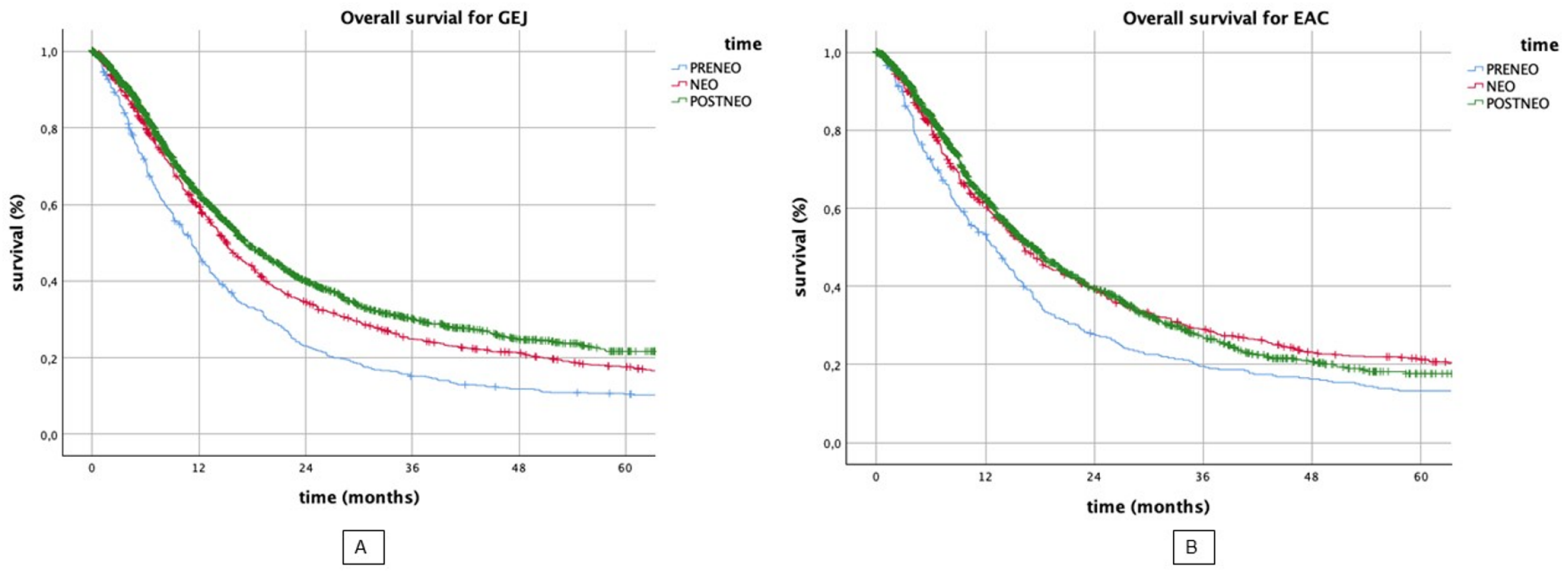
Kaplan-Meier Plot for overall survival of GEJ and EAC over time

The analysis of the stage independent overall survival of the entire population of patients with EAC and GEJ in the three eras PRE-NEO, NEO and POST-NEO revealed that in fact survival improved steadily over time (*p* < 0.001, respectively) (Fig. 1). PRE-NEO is associated with the shortest OS among all time periods. For example, the median OS increased from 11 months (PRE-NEO) over 15 months (NEO) to 17 months (POST-NEO) in GEJ. Similar trends were observed in EAC (PRE-NEO: 13 months; NEO: 16 months; POST-NEO: 17 months).

### Changes in treatment intentions for GEJ and EAC over time

**Fig. 2:**
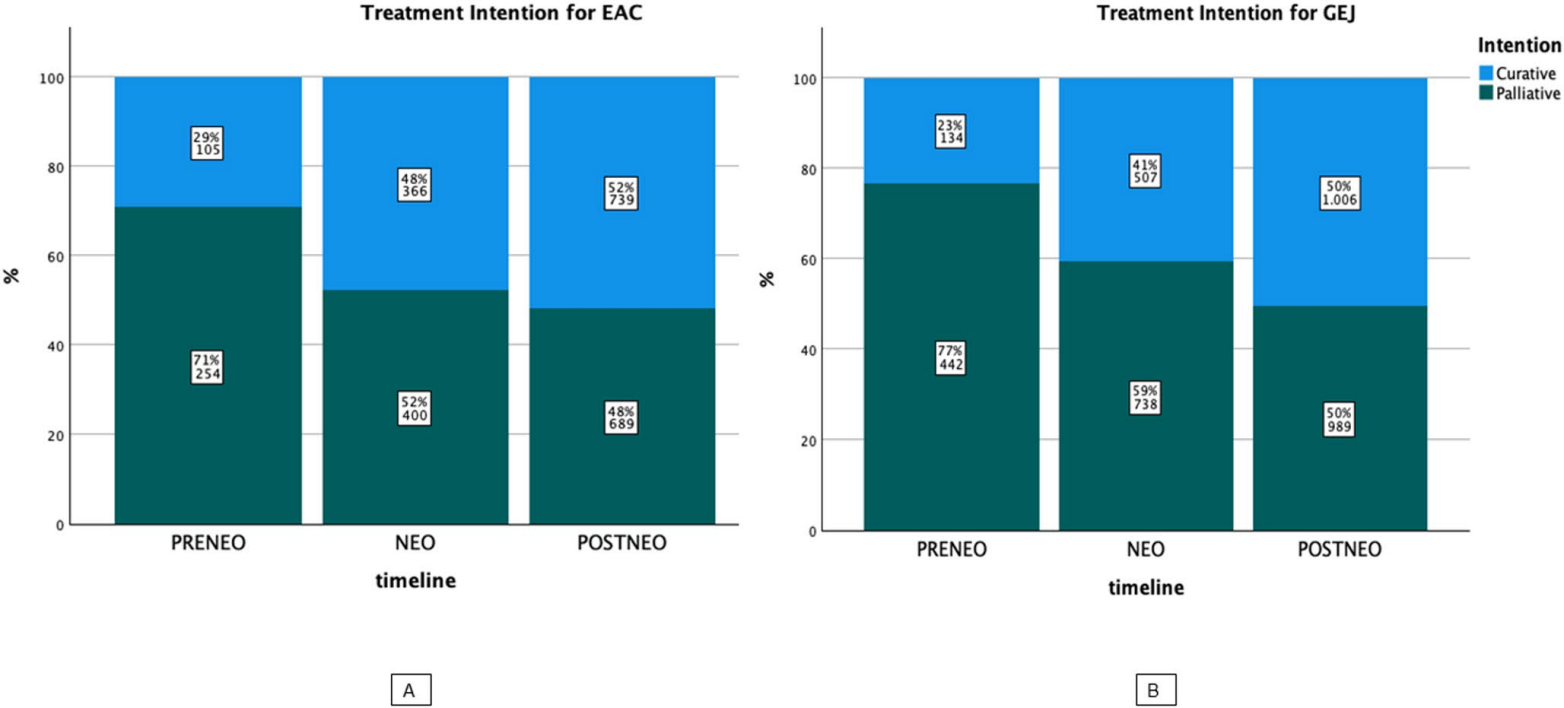
Distribution of treatment intention in EAC and GEJ over time

The treatment intentions (curative vs. palliative) changed remarkably over time for both EAC and GEJ (*p* < 0.001, respectively) (Fig. 2). For EAC, the reported proportion of curative intended treatments increased from 29% (PRE-NEO) to 52% (POST-NEO). Similar pattern were observed in GEJ with curative treatment increasing from 23% (PRE-NEO) to 50% (POST-NEO). This changes occurred in parallel in hand with the changes in overall survival in the respective time periods.

### Changes in tumor stages for GEJ and EAC over time

**Fig. 3:**
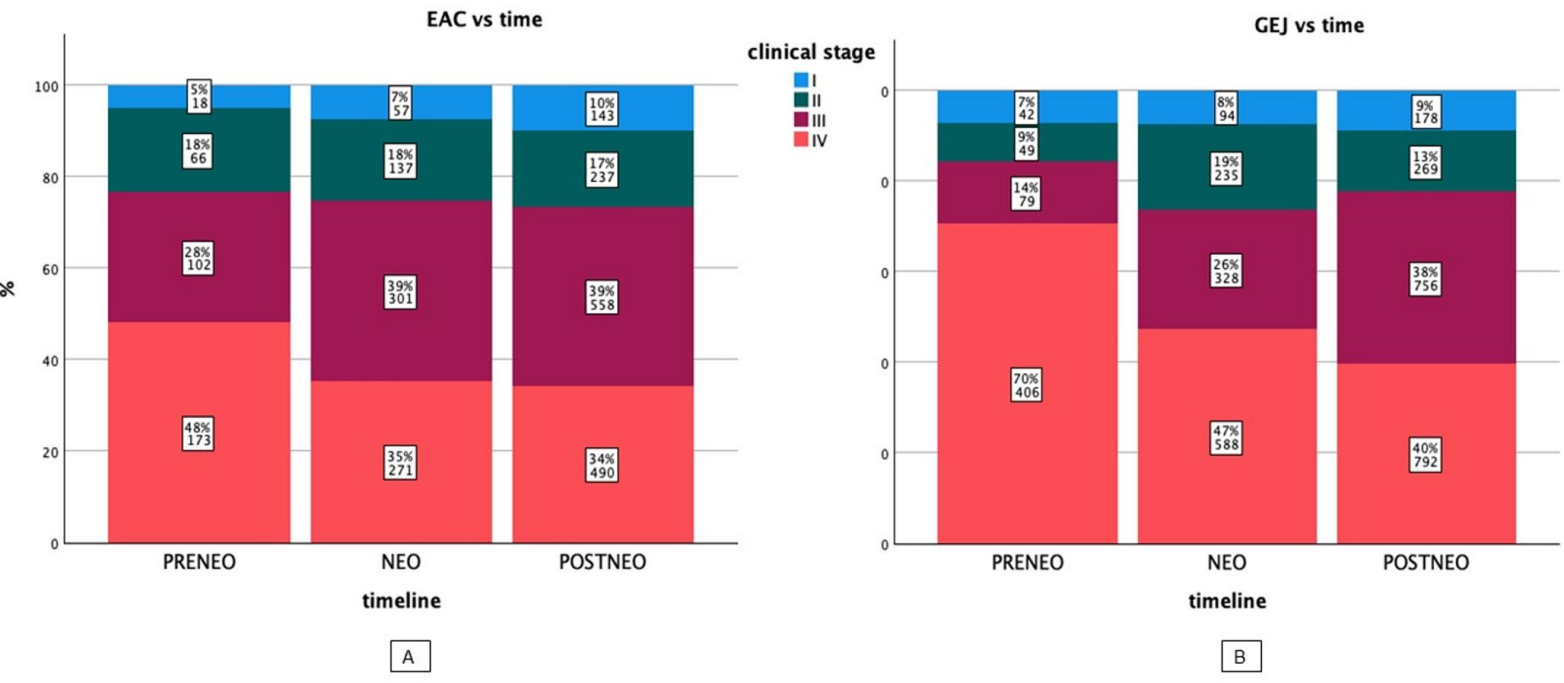
Distribution of cTNM stages in EAC and GEJ over time

As one potential confounder for better survival or changes in treatment intentions over time might lie in better diagnostics with detection of tumors in earlier stages, we first analysed the distribution UICC stages over time. While the proportion of patients diagnosed with stage I and II disease remained fairly similar over the time for both GEJ and EAC, we found indeed a significant increase in patients with documented stage III disease over time from 14% (PRE-NEO) to 38% (POST-NEO) for example in GEJ. However, for EAC in stage III the differences were notably lower with 28% (PRE-NEO) to 39% (POST-NEO). On the other hand, proportion of documented stage IV disease decreased continuously from 70% to 40% in the same periods for GEJ. Again, these changes were less pronounced in EAC stage IV from 48% to 34% (Fig. 3).

### Changes for curative therapy options in GEJ and EAC over time and their effect on overall survival

In this subgroup analysis we included only patients with curative treatment intentions (*n* = 2857). The analyzed treatment strategies included: primary surgery, multimodal therapy and definitive radiochemotherapy. Multimodal therapy included perioperative chemotherapy as well as neoadjuvant radiochemotherapy followed by tumor resection.

Overall, the curative treatment strategies changed significantly over time for both entities (*p* < 0.001, respectively). For example, in EAC both, perioperative chemotherapy (5% PRENEO to 31% POSTNEO) and neoadjuvant RCT + surgery (5% PRENEO to 13% POSTNEO) increased over time. Patients who underwent surgery alone decreased for both entities, for example in EAC (PRENEO 76% to POSTNEO 52%). In comparison, definitive radiochemotherapy decreased over time (15% PRENEO to 4% POSTNEO). Regarding multimodal therapy for GEJ similar trends were observed in the same time periods (Fig. S3).

### Overall survival in UICC stage II

**Fig. 4:**
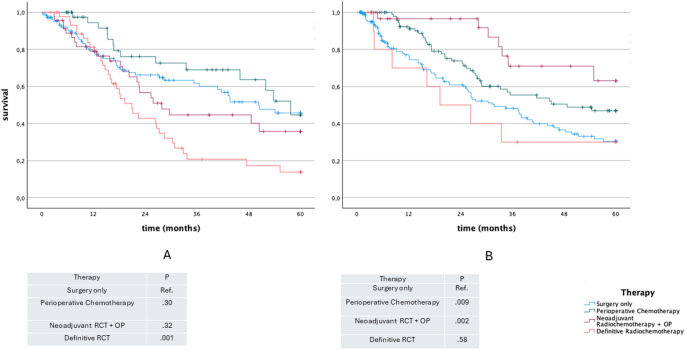
Kaplan-Meier plot for overall survival according to curative therapy UICC II

For GEJ tumors in Stage II, both neoadjuvant radiochemotherapy followed by surgery (mean 50.6 months; 95% CI: 44.4–56.8; *p* = 0.002) and perioperative chemotherapy followed by surgery (mean 41.3 months; 95% CI: 36.9–45.7; *p* = 0.009) were associated with longer overall survival compared to surgery alone (mean 33.7 months; 95% CI: 29.7–37.7). No statistically significant difference was observed between the two multimodal treatment strategies (*p* = 0.078) (Fig. 4).

In contrast, in Stage II EAC, neither perioperative chemotherapy (*p* = 0.31) nor neoadjuvant radiochemotherapy followed by surgery (*p* = 0.32) was significantly associated with improved overall survival compared to surgery alone (Fig. 4).

### Overall survival in UICC stage III

**Fig. 5:**
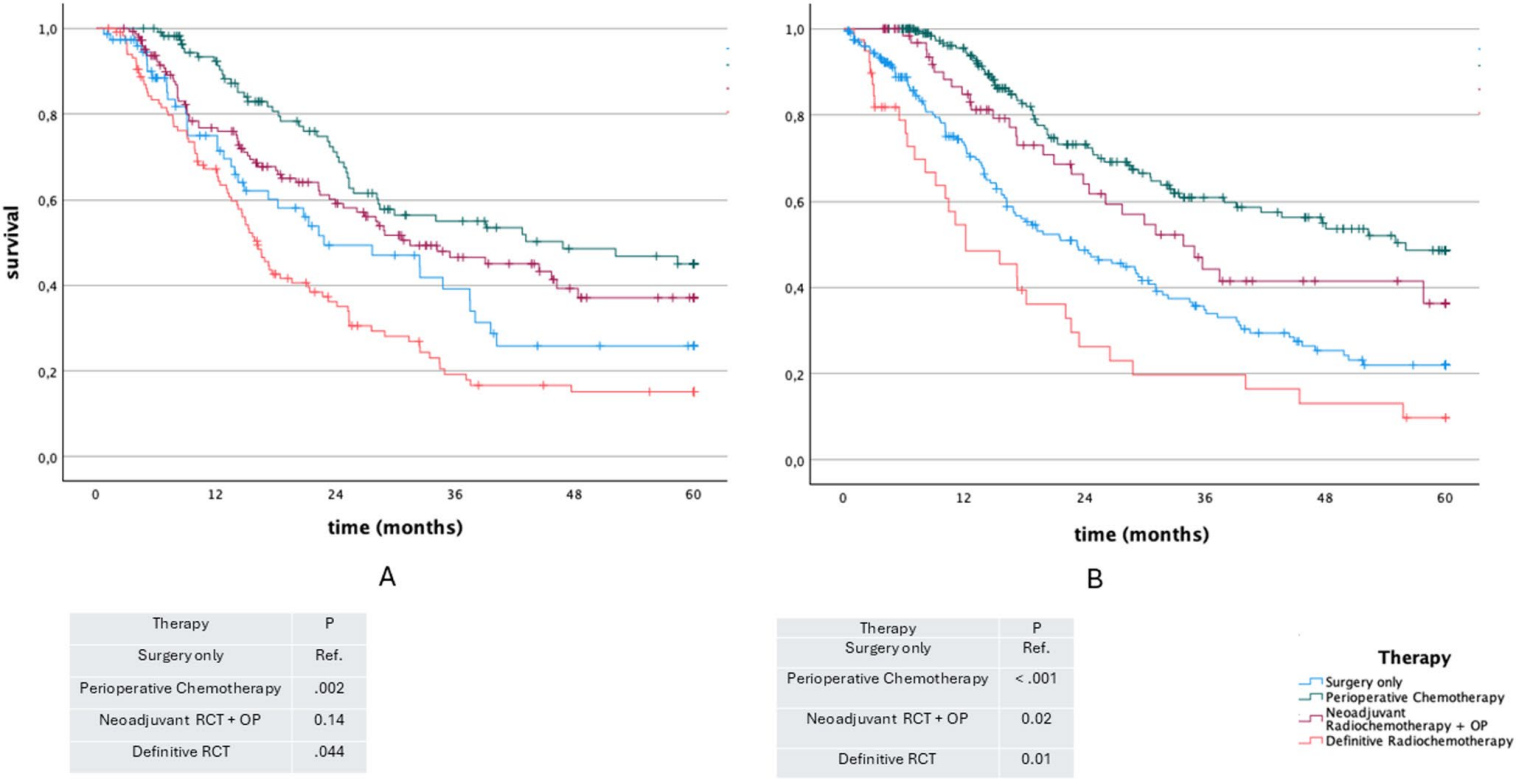
Kaplan-Meier plot for overall survival according to curative therapy UICC III

In GEJ, surgery only (mean 28.8 months; 95% CI: 25.4–32.1) was associated with a significant poorer OS compared to perioperative chemotherapy (mean 42.8 months; 95% CI: 39.5–46.0) (*p* < 0.001) and neoadjuvant radiochemotherapy + surgery (mean 37.0 months; 95% CI: 31.2–42.8) (*p* = 0.018). Although perioperative chemotherapy tended to be associated with longer OS, it did not reach statistical significance when compared to neoadjuvant radiochemotherapy + surgery (*p* = 0.064). Furthermore, definitive radiochemotherapy (mean 20.16 months; 95% CI: 13.9–26.4) was associated with a significant lower OS compared to surgery only (*p* = 0.01), perioperative chemotherapy (*p* < 0.001) and neoadjuvant radiochemotherapy + surgery (*p* < 0.001) (Fig. 5).

Considering the overall survival in EAC in stage III, surgery only (mean 29.8 months; 95% CI: 24.2–35.5) was associated with a significant lower OS compared to perioperative chemotherapy (mean 40.5 months; 95% CI: 36.3–44.7) (*p* = 0.002). However, there was no significance comparing surgery only to neoadjuvant radiochemotherapy followed by sugery (mean 35.1 months; 95% CI: 36.3–44.7) (*p* = 0.141). Although perioperative chemotherapy tended to show longer OS compared to neoadjuvant radiochemotherapy (mean 35.1 months; 95% CI: 36.3–44.7) followed by sugery. However, statistical significance was not reached (*p* = 0.07) (Fig. 5).

### Multivariable analysis for overall survival

In multivariable Cox regression analysis adjusting for age group, sex, tumor location, clinical stage, treatment category, and treatment era, multimodal therapy remained independently associated with improved overall survival compared with surgery alone (HR 0.767, 95% CI 0.675–0.872; *p* = 0.001). Higher clinical stage was associated with increased mortality risk, whereas treatment era was not independently associated with survival after adjustment (Table S1).

### Overall survival in stage IV disease

A subgroup analysis was performed for patients with Stage IV who underwent curative intended therapy. In GEJ, only perioperative Chemotherapy (mean 36.9 months; 95% CI: 27.7–46.1) was associated with longer overall survival compared to surgery only (mean 24.0 months; 95% CI: 17.8–30.2) (*p* = 0.025) (Fig. S4). However, there was no survival benefit for surgery only, or radiochemotherapy follow by surgery when compared to definitve radiochemotherapy. In EAC, There was no significant association with improved overall survival for other treatment modalities when compared to definitive radiochemotherapy.

## Discussion

In this population-based study, a comprehensive analysis was conducted to demonstrate the evolution of treatment strategies concerning adenocarcinoma of the esophagus (EAC) and gastroesophageal junction (GEJ) in Germany. Here, we analyzed the impact of these changes on patients’ survival over the past two decades, using data derived from 24 clinical registries provided by the German Cancer Registry Group of the Society of German Tumor Centers - Network for Care, Quality, and Research in Oncology (ADT) in Germany.

The study’s findings indicate a rise in the reported frequency of both EAC and GEJ, with respective increments of 2.3 and 1.8 folds over the period of two decades. This surge was observed from 274 patients with EAC and 535 patients with GEJ reported in 2000 to 648 and 958 patients reported in 2018 in the same registries. Similar trends were previously documented by the Robert-Koch-Institute in 2017 [7]. This improvements in overall survival likely reflect a combination of evolving treatment strategies, changes in staging documentation, and broader improvements in oncologic care in Germany during this period. This surge can be explained multifactorial, on one side it could be the result of increased reporting and improved documentation of the treatment providers to their corresponding local cancer registries but also an increased awareness and changes in diagnostic work-up over time. These observations may suggest a potential shift towards earlier tumor stages (I-III) and a reduction in stage IV for both EAC and GEJ across the time periods PRE-NEO 2000–2006, NEO 2007–2012, and POST-NEO 2013–2018. This findings are, however, best interpreted as contextual findings rather than proof of earlier detection.

Furthermore, our study revealed a considerable improvement in overall survival for patients afflicted with both EAC and GEJ over time. Median overall survival significantly increased for GEJ tumors from 11 to 17 months and for EAC from 13 to 17 months. The underlying factors contributing to this effect are likely multifaceted, including advancements in endoscopic and surgical techniques, as well as the implementation of new chemotherapeutic agents and multimodal treatment strategies.

Since the beginning of the new millennium, the treatment strategies of esophageal and GEJ tumors improved drastically, particularly after the conduction of several landmark trials such as the MRC (OEO2) Trial in 2002 [8], the MAGIC Trial in 2006 [9], the CROSS Trial in 2012 [10], and the FLOT-4 Trial in 2019 [11]. The impact of these studies led to a substantial upsurge in multimodal therapy in Germany, which increased from 8% in 2000–2006 to 19% in 2007–2012 and 25% in 2013–2018. Similar trends were also observed in a population-based analysis from the Netherlands [12].

The rationale behind multimodal treatment extends beyond improving resectability and achieving complete pathological response; it also aims to address micrometastasis, prevent early disease relapse, and reduce distant metastasis [2].

Furthermore, we undertook an analysis of changes in treatment strategies and intentions for each clinical stage. This resulted in an increase in curative-intended therapy for both entities over the study period. Specifically, the percentage of patients with EAC and GEJ receiving curative-intended therapy rose significantly from 29% to 23% in 2000–2007 to 52% and 50% in 2013–2018, respectively (Fig. 3). These changes were predominantly observed in clinical stage II and stage III, leading to an overall improvement in survival for the entire cohort. While a population-based study from the Netherlands noted a decrease in “No Treatment” or “Other Therapies” in non-metastatic EAC over 18 years, a stage-dependent analysis had not been conducted previously [12].

According to the current S3-German Guidelines, patients diagnosed with locally advanced disease (cT3 or cN+) which encompasses all patients with stage III and nodal positive patients in stage II are recommended to undergo radiochemotherapy or perioperative chemotherapy [1]. These guidelines are broadly consistent with results in our study, hereby multimodal therapy was associated with longer overall survival for all stage III patients, irrespective of tumor location. Additionally, patients with GEJ tumors at stage II demonstrated a longer overall survival.

Our study showed in the univariable analysis longer overall survivals in perioperative chemotherapy compared to surgery only especially in GEJ and EAC for UICC III. However, no statistical significance was proven among perioperative Chemotherapy and neoadjuvant RCT + surgery. This marginally non-significant result in favour for perioperative Chemotherapy could be due to the fact that there was no differentiation between the two perioperative chemotherapy protocols of ECF and FLOT in the presented study. Any numerical differences should therefore be interpreted cautiously, as treatment allocation may also reflect tumor characteristics, center preference, and patient selection. In order to further elucidate this issue, a second study of our research group is being conducted to differentiate a survival benefit between the perioperative chemotherapy protocol FLOT and neoadjuvant RCT + surgery (CROSS).

In Europe, perioperative chemotherapy using the FLOT-Protocol (5-FU, Leucovorin, Oxaliplatin, and Docetaxel) [11] or the CROSS-Protocol, involving chemoradiotherapy with carboplatin, paclitaxel, and radiation at 41 Gy followed by surgery [10], are widely adopted multimodal regimes [11]. The results of the ESOPEC trial have shown superiority of FLOT compared to CROSS [13]. Our study demonstrated comparable overall survival outcomes with perioperative chemotherapy or chemoradiotherapy for both esophageal adenocarcinoma (EAC) and GEJ tumors, at cTNM Stage II and III. Although the exact chemotherapeutic agents are not provided in our study (FLOT vs. ECF). These findings are in concurrence with results from the NEO-AEGSIS trial, which compared modified MAGIC or FLOT vs. CROSS protocols and did not reveal definitive evidence favoring one procedure over the other in terms of tumor recurrence [14]. A large retrospective study (*n* = 578) comparing both protocols reported similar findings [15].

Several limitations should be considered when interpreting the results of this study, mostly being related to the retrospective design and registry-based analysis. Since our data were derived from 24 clinical cancer registries, possible inconsistency in reporting likely existed. The Sensitivity analyses comparing patients with and without documented cTNM staging demonstrated statistically significant but modest and inconsistent survival differences across time periods. These findings suggest that missing staging data likely reflect heterogeneous clinical and documentation contexts rather than a uniformly distinct prognostic subgroup. However, Missing cTNM documentation (around 40%) remains a relevant limitation of the present analysis. Although the sensitivity analysis was overall reassuring, incomplete staging may still have influenced stage-specific estimates, and these findings should therefore be interpreted with appropriate caution. Moreover, this study did not take the histological stage after resection into consideration, since the treatment strategies were determined according to the cTNM stage preoperatively. Moreover, information regarding the postoperative complications, the number of administrated cycles or the exact chemotherapeutic regimes or the use biologicals were not included in this study. Despite these drawbacks, our large sample size provides analytic power and provide support for guideline based multimodal therapy in EAC and GEJ tumors.

## Conclusion

The number of cases of adenocarcinoma of the esophagus and gastroesophageal junction registered in German cancer registries has risen over the past two decades. Overall survival improved over time, likely reflecting a combination of changes in stage distribution, treatment allocation, and broader advances in multimodal care. Multimodal therapy was independently associated with improved overall survival in cTNM Stage III disease and Stage II GEJ tumors; these findings are consistent with current guideline-based treatment strategies.

## Supplementary Information

Below is the link to the electronic supplementary material.

Supplementary Material 1 (DOCX 2.04 MB)

## Data Availability

No datasets were generated or analysed during the current study.
