# Supplementary material for: Effect of multimodal therapy in the treatment of adenocarcinoma of the esophagus and gastroesophageal junction : a population-based analysis from the German Clinical Cancer Registry Group
Source: Langenbecks Arch Surg. 2026 May 29;411(1):148. doi: 10.1007/s00423-026-04066-7 (PMC13222164; doi:10.1007/s00423-026-04066-7)
Supplement: Supplementary file 1 — Supplementary Material 1 (DOCX 2.04 MB) [file 423_2026_4066_MOESM1_ESM.docx]

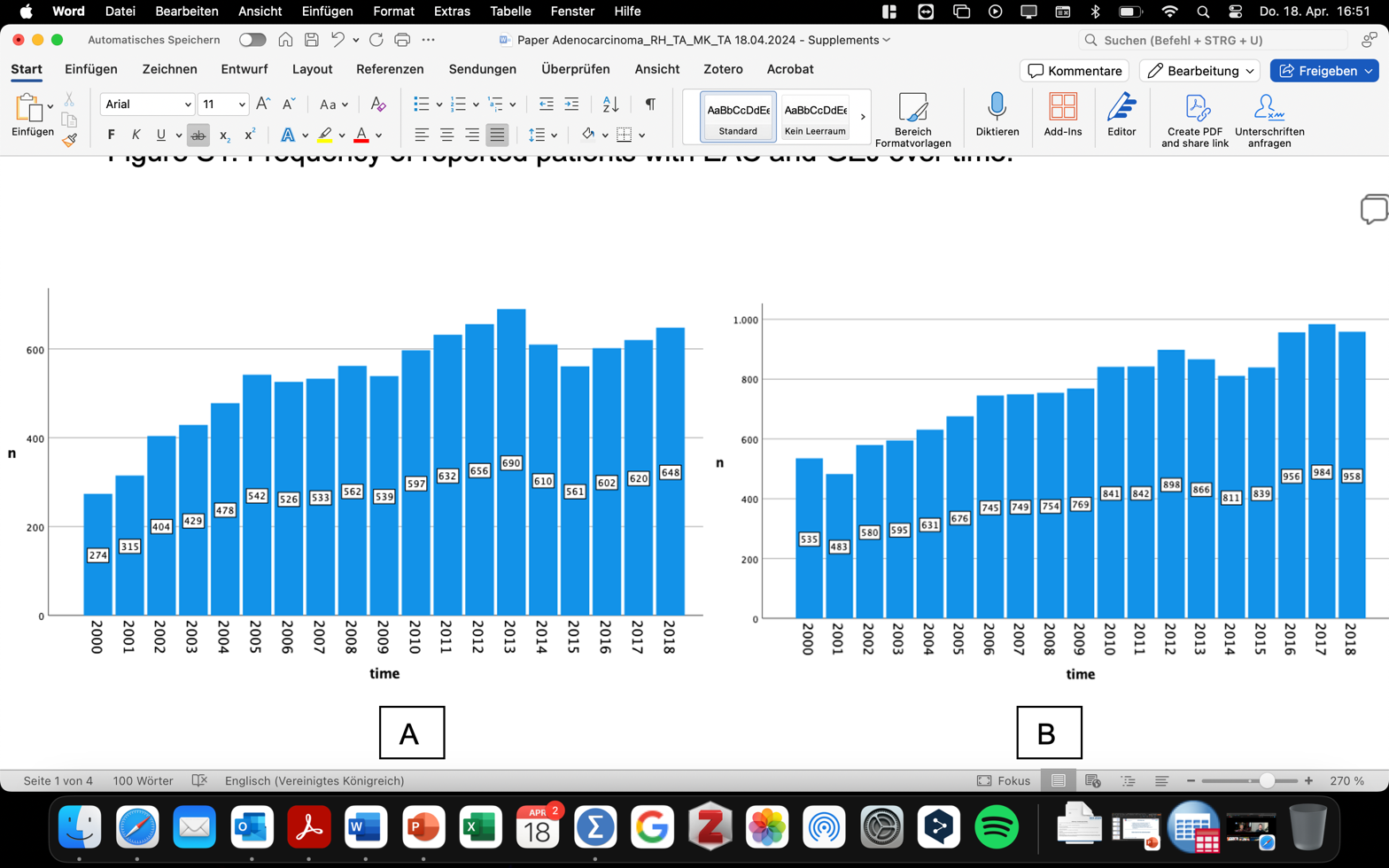
Figure S1: Frequency of reported patients with EAC and GEJ over time.

B

A

Legend: Figure A; EAC, Figure B; GEJ

Figure S2: Comparison sex to organ


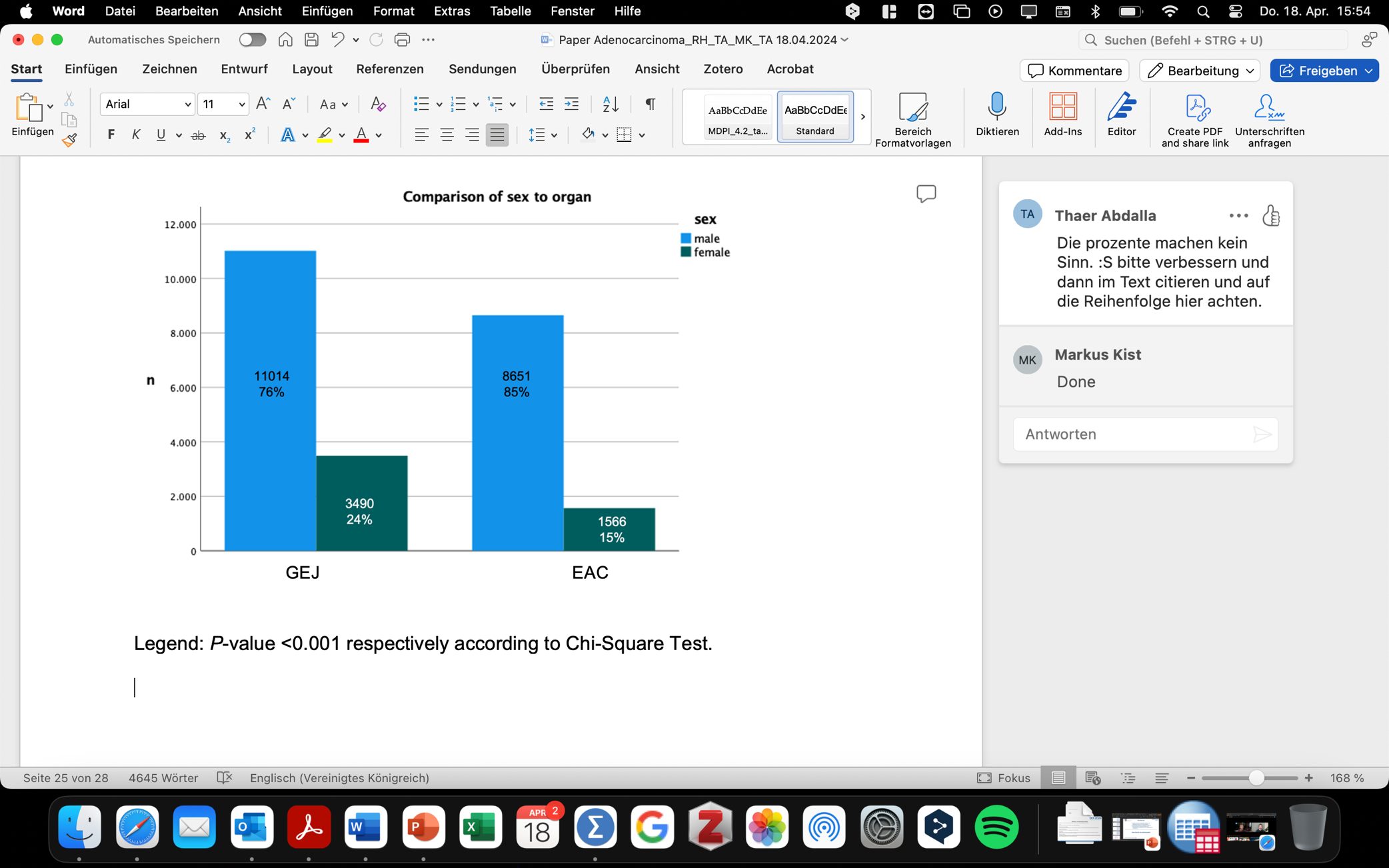


A

Legend: Figure A; GEJ and EAC


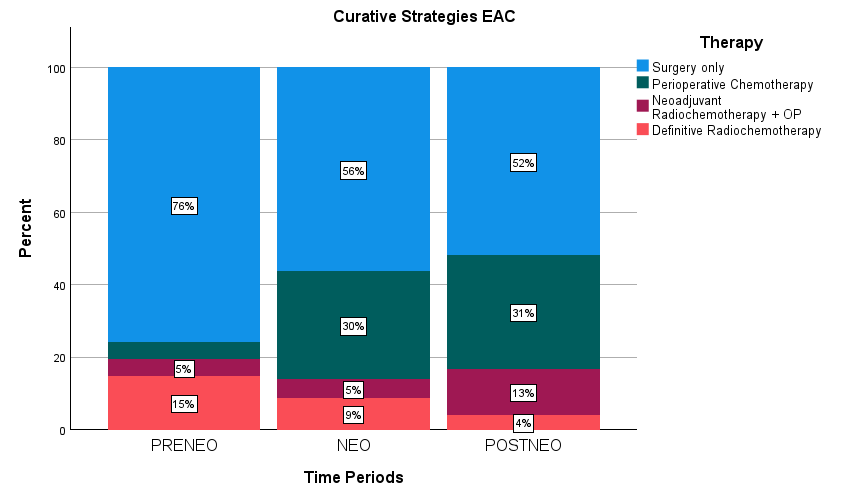

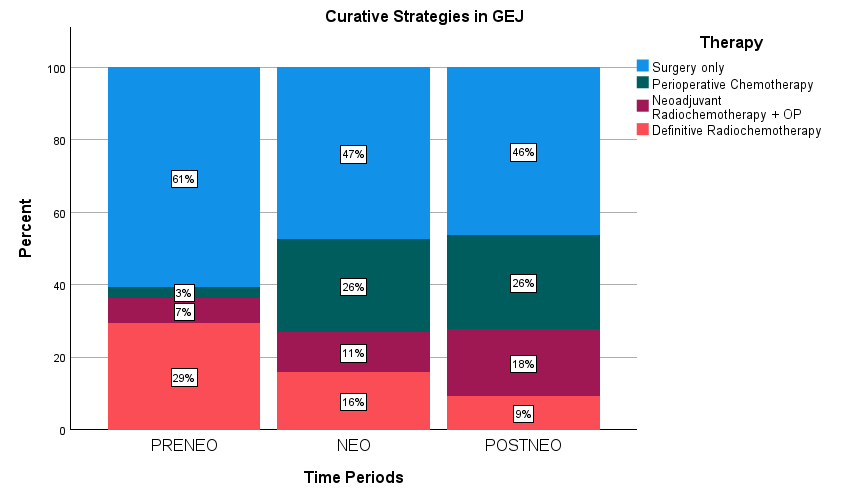
Figure S3: Distribution of curative treatment strategies in GEJ and EAC over time

B

A

Legend: Figure A; EAC, Figure B; GEJ, PRENEO (2000-2006), NEO (2007-2012), POSTNEO (2013-2018). *P*-value <0.001 respectively according to Chi-Square Test.


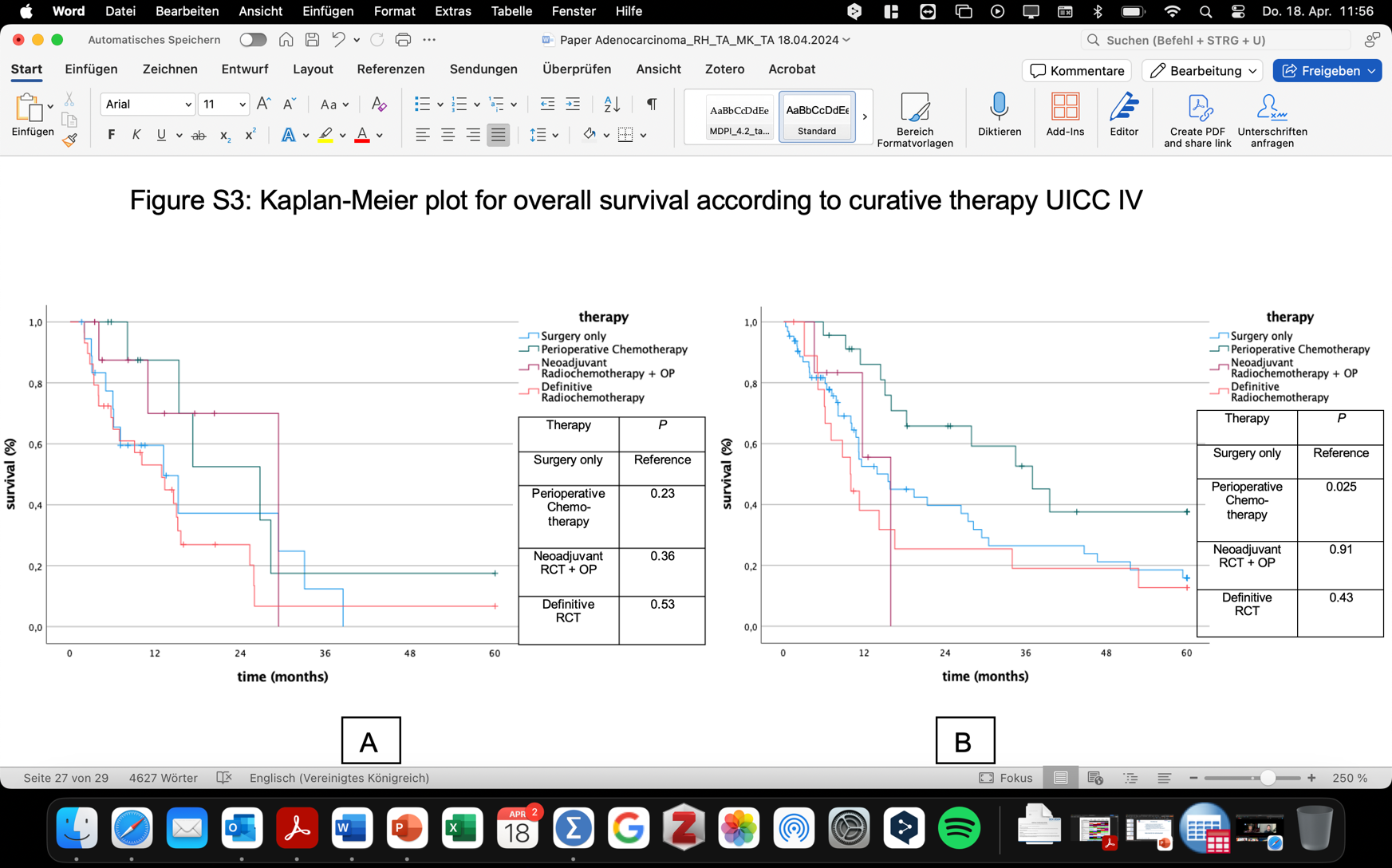
Figure S4: Kaplan-Meier plot for overall survival according to curative therapy UICC IV

B

A

Legend: Figure A: EAC, Figure B: GEJ, P-value <0.0001 (respectively) according to logrank test.

Figure S5. Sensitivity survival analysis according to documented staging


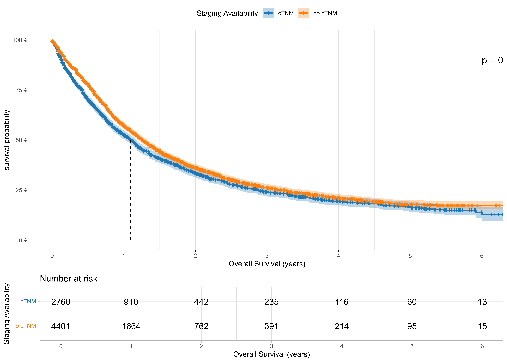

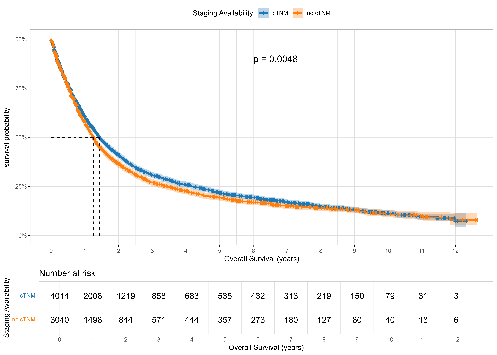

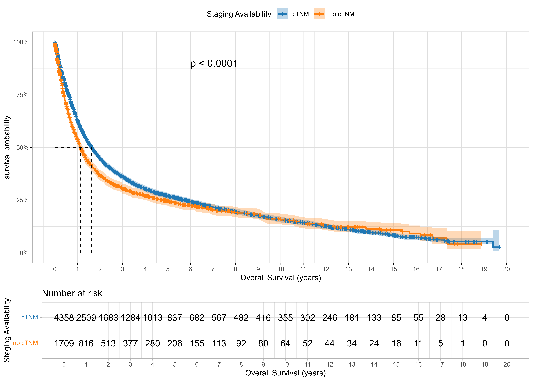


POSTNEO

NEO

PRENEO

Legend: Kaplan–Meier curves for overall survival comparing patients with documented cTNM staging and patients without documented staging across the three predefined time periods: PRE-NEO (2000–2006), NEO (2007–2012), and POST-NEO (2013–2018), P-value =0., P-value <0.001, and P-value =0.001 respectively. Survival distributions were compared using the log-rank test. The analysis was performed to assess the potential impact of missing staging information on survival estimates.

Table S1. Multivariable Cox proportional hazards regression analysis for overall survival.

|  | Hazard-Ratio | 95% CI | p-value |
| --- | --- | --- | --- |
| Sex | 1.027 | 0.945 - 1.116 | 0.537 |
| Age Groups | 1.363 | 1.278 - 1.453 | 0.001 |
| Location | 1.051 | 0.985 - 1.122 | 0.135 |
| Therapy  OP alone  multimodale Therapy  Definitive R/C  Palliative | -  0.767  1.561  2.022 | -  0.675 – 0.872  1.318 – 1.850  1.805 – 2.265 | reference  0.001  0.001  0.001 |
| Stages  Stage I  Stage II  Stage III  Stage IV | -  1.330  1.647  2.717 | -  1.118 – 1.582  1.396 – 1.944  2.295 – 3.216 | reference  0.001  0.001  0.001 |
| Timline  2000-2006  2007-2013  2013-2018 | -  1.050  1.036 | -  0.962 – 1.147  0.965 – 1.113 | reference  0.273  0.326 |

Legend: The model was adjusted for age group, sex, tumor location (EAC vs. GEJ), clinical UICC stage (I–IV), treatment category (surgery alone [reference], multimodal therapy, definitive radiochemotherapy, palliative therapy), and treatment era (PRE-NEO [reference], NEO, POST-NEO). Hazard ratios (HR) with 95% confidence intervals (CI) are reported. Overall survival was calculated from date of diagnosis to death or last follow-up.
